# ASO Author Reflections: Sex-Associated Aspects of Referral, Resectability, and Survival in Patients with Colorectal Liver Metastases

**DOI:** 10.1245/s10434-025-18904-3

**Published:** 2025-12-15

**Authors:** Martina Nebbia, Jennie Engstrand

**Affiliations:** Division of Surgery and Oncology, Department of Clinical Science, Intervention and Technology, Karolinska InstitutetKarolinska University Hospital, Stockholm, Sweden

## Past

Sex-related differences in colorectal cancer biology and outcomes have been increasingly recognized, with women often presenting with right-sided primaries, older age, and distinct molecular profiles such as higher *BRAF* or *KRAS* mutation rates.^1,2^ Earlier Swedish registry studies raised concerns that women with colorectal liver metastases (CRLM) were less likely to undergo hepatic resection than men, even after adjustment for disease stage.^3^ Whether these disparities reflected true biological differences or inequities in referral and treatment allocation remained unclear. Given the pivotal role of multidisciplinary team (MDT) assessment in modern CRLM management,^4^ we sought to determine whether standardized MDT evaluation could mitigate sex-related disparities in access to curative-intent therapy.

## Present

In our population-based study of 1207 patients with CRLM referred to the Stockholm regional liver MDT between 2013 and 2021, we observed no sex-associated differences in resectability, treatment allocation, or survival.^5^ Women accounted for 39% of referred cases, were slightly younger, and had a higher prevalence of *KRAS* mutations, yet the extent of liver and extrahepatic disease was comparable between sexes. Curative liver-directed interventions were performed in 59% of women and 62% of men (*p* = 0.219), with no difference in postoperative outcomes. Across all subgroups, including resected, palliative, and liver-only disease, overall survival was similar, and multivariable Cox regression confirmed that sex was not an independent predictor of outcome (5). These results suggest that structured MDT referral pathways can neutralize previously reported sex-related disparities in treatment access and outcomes.

## Future

Our findings underscore the importance of mandatory referral of all patients with metastatic colorectal cancer to specialized hepatobiliary MDTs, ensuring equitable and biology-driven treatment. Systematic, centralized assessment by liver surgeons and oncologists appears critical to achieving treatment parity between sexes. Future investigations should explore earlier steps in the care pathway, particularly referral practices from diagnosing oncologists and colorectal teams, to identify residual barriers. Integrating molecular and early-onset data may further elucidate subtle sex-based biological differences and guide personalized therapeutic strategies for patients with CRLM.

## References

[1] Kim SE, Paik H-Y, Yoon H, et al. Sex- and gender-specific disparities in colorectal cancer risk. *World J Gastroenterol*. 2015;21(17):5167–75. 25954090 10.3748/wjg.v21.i17.5167 PMC4419057

[2] Papachristodoulou A, van der Valk MJM, van der Geest LGM, et al. Sex-specific differences in patients undergoing surgery for colorectal liver metastases. *HPB (Oxford)*. 2021;23(6):872–9.

[3] Nordenvall C, Nilsson PJ, Mahteme H, et al. Sex disparities in colorectal cancer management and outcome in a population-based cohort. *J Surg Oncol*. 2019;120(6):1074–82.

[4] Almlöv K, Arbman G, Björnsson B, et al. Assessment by a multidisciplinary team conference affects treatment strategy and overall survival in patients with synchronous colorectal liver metastases. *HPB (Oxford)*. 2024;26(9):1131–40. 38849249 10.1016/j.hpb.2024.05.008

[5] Nebbia M, Villard C, Gilg S, Sparrelid E, Gerling M, Engstrand J. Sex-associated aspects of referral, resectability, and survival in patients with colorectal liver metastases: a population-based study of 1,207 MDT-referred cases. *Ann Surg Oncol*. 2025. (10.1245/s10434-025-18745-0). 10.1245/s10434-025-18919-w 41483272

